# Two Unusual Aspects of Posterior Reversible Encephalopathy Syndrome Mimicking Primary and Secondary Brain Tumor Lesions

**DOI:** 10.1155/2015/456217

**Published:** 2015-10-26

**Authors:** Mazamaesso Tchaou, Nicoleta Modruz, Lama K. Agoda-Koussema, Anthony Michelot, Samer Naffa, Véronique Jeudy, Raymond Kaczmarek

**Affiliations:** ^1^Department of Radiology, Lomé Teaching Hospital, University of Lomé, Lomé, Togo; ^2^Department of Radiology, Cornouaille Intercommunal Hospital, 29000 Quimper, France

## Abstract

The posterior reversible encephalopathy syndrome (PRES) is a rare clinical-radiological entity well described with typical clinical and radiological manifestations. Atypical presentation, especially in imaging, exists. The authors report here two cases of posterior reversible encephalopathy in which imaging aspects were atypical, mimicking, in the first case, hemorrhagic cerebral metastasis of cholangiocarcinoma and, in the second case, a brain tumor. The diagnosis has been retrospectively rectified due to clinical and radiological outcome.

## 1. Introduction

Described for the first time in 1996 by Hinchey et al. [1], the posterior reversible encephalopathy syndrome (PRES) is a clinical-radiological entity combining clinical manifestations such as seizures, headache, visual disturbances, consciousness impairment, nausea/vomiting, and focal neurological signs [1–3]. In imaging, posterior reversible encephalopathy is typically characterized by partially or completely reversible bilateral and symmetrical subcortical vasogenic edema preferentially affecting the posterior regions of the brain [1]. In addition to its typical appearance, there is a great variability both in clinical manifestations and in the imaging aspects of this syndrome.

We report here two cases of posterior reversible encephalopathy in which imaging aspects were atypical, mimicking, in the first case, hemorrhagic cerebral metastasis of cholangiocarcinoma and, in the second case, a brain tumor because of its unilateral localization.

## 2. Cases Presentation

### 2.1. Case 1

A 71-year-old male patient with known hypertension and history of myocardial infarction that required a double bypass surgery 38 years ago, cardiac arrhythmias due to atrial fibrillation (AF), and an old thrombocytopenia has been urgently admitted for onset of neurological disorders manifested as visual disturbances. Clinical examination found an acute hypertensive episode with an elevated blood pressure of 180/110 mmHg.

**Figure 1:**
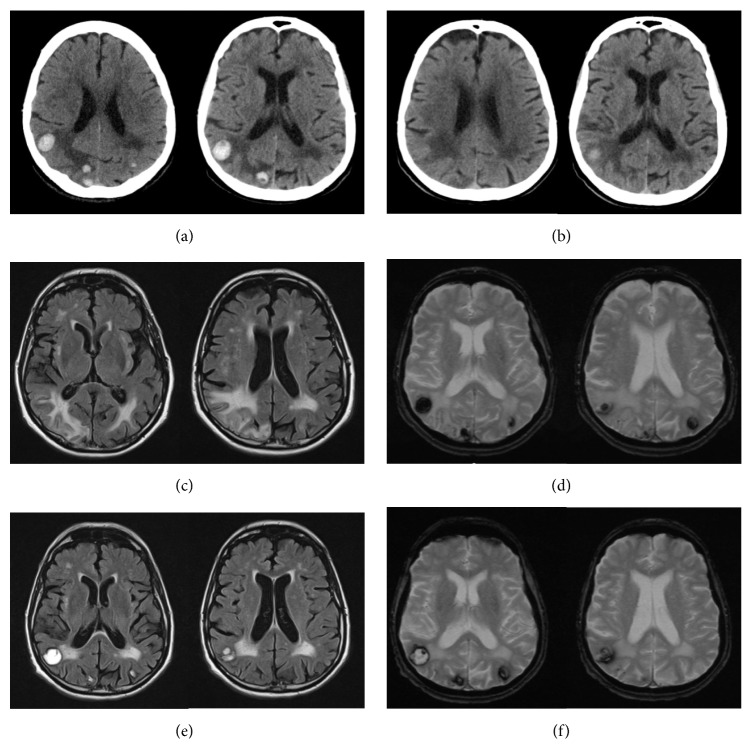
Patient 1: noncontrast brain CT-scan showing occipital bilateral hyperdense nodules, predominantly on the right side, surrounded by edema, corresponding to brain hematomas (a). Noncontrast brain CT-scan 18 days later showing regression of the size and number of hematomas with a decrease in the degree of the surrounding edema (b). MRI-scan 6 weeks later, axial Fluid Attenuated Inversion Recovery, FLAIR (c), and Gradient Echo, GRE (d), weighted sequences showing a marked susceptibility effect confirming the haemorrhagic lesions and the presence of surrounding edema. Follow-up MRI-scan 4 months later with same sequences, showing regression of the size and number of hematomas with increase in the size of the vasogenic edema in the left occipital lobe (e and f).

A brain noncontrast CT-scan was performed and found bilateral occipital nodular hematomas, predominantly in the right hemisphere, surrounded by edematous hypodense white matter (Figure 1(a)). Faced with this aspect, the hypothesis of hemorrhagic brain metastases secondary to a neoplastic tumor was raised as first line. The search for a primary neoplastic lesion by performing thoracic-abdominal-pelvic CT-scan revealed dilatation of the intrahepatic bile ducts upstream of a hilar hepatic lesion—which was proved to be a cholangiocarcinoma. A follow-up brain CT-scan performed 18 days later, following the disappearance of the visual disturbances, with the only treatment to control the hypertension, found a partial regression of the lesions (Figure 1(b)).

A brain MRI-scan performed 6 weeks later (Figures 1(c) and 1(d)) and another follow-up brain MRI-scan performed 4 months later (Figures 1(e) and 1(f)) confirmed the hemorrhagic nature and evolution of the lesions, the absence of new hemorrhagic lesions, and the increase in size of the vasogenic edema in the left occipital area. No abnormal brain enhancement was proven.

Given the evolution of radiological images and the disappearance of neurological disorders in the absence of a specific treatment except the one for controlling the hypertension and the chemotherapy for his cholangiocarcinoma, the final diagnosis was intracerebral hemorrhagic complicated form of PRES. For his hepatobiliary tumor he had initially benefited from chemotherapy by GEMOX that allowed stabilizing the lesions and in a second phase he had a right hepatectomy.

### 2.2. Case 2

**Figure 2:**
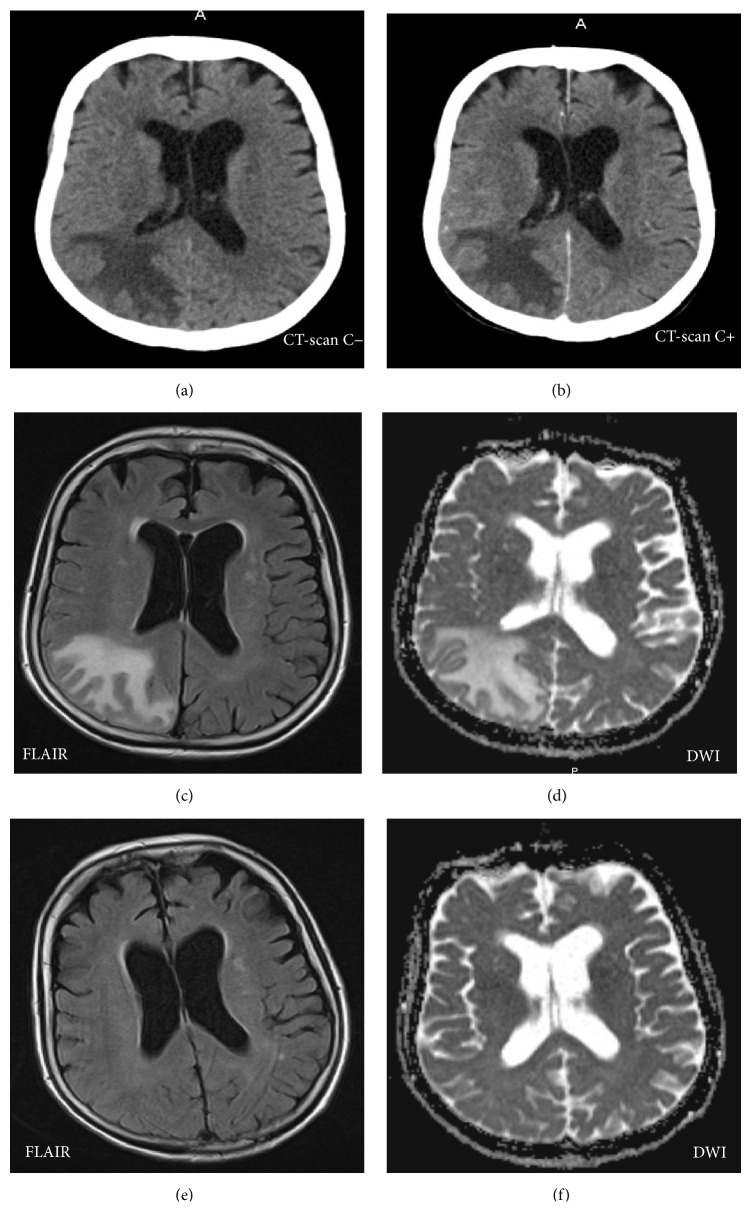
Patient 2: noncontrast brain CT-scan (a) showing a diffuse area of unilateral hypoattenuation in the right white matter in the parietooccipital lobes with a discrete mass effect on the occipital horn of the lateral ventricle, without contrast enhancement (b), in favour of a space occupying lesion of the brain. MRI-scan, axial FLAIR (c) and Diffusion Weighted Imaging, DWI (d), showing an area of FLAIR hyperintensity without diffusion restriction and without contrast enhancement with the same localization as on the CT-scan corresponding to a vasogenic cerebral edema. Follow-up MRI-scan one month later, axial FLAIR (e) and DWI (f), showing the disappearance of the parietooccipital hyperintense area corresponding to a vasogenic cerebral edema, confirming PRES.

A 77-year-old woman was admitted in emergency room for persistent headache in a context of high blood pressure at 175/115 mmHg. Noncontrast and contrast brain CT-scans were performed and showed a diffuse area of unilateral hypoattenuation in the right white matter in the parietooccipital lobes finger in glove appearance with a discrete mass effect on the occipital horn of the lateral ventricle, without contrast enhancement, in favour of a space occupying lesion of the brain (Figures 2(a) and 2(b)). The brain MRI-scan confirmed the presence of a vasogenic edema in the right white matter in the parietooccipital lobes (Figures 2(c) and 2(d)). No contrast enhancement was proved. The hypothesis of a low grade glioma was raised. A new brain follow-up MRI-scan performed a month later after the control of blood pressure and the disappearance of headache (Figures 2(e) and 2(f)) showed the disappearance of the parietooccipital hyperintense area, confirming a PRES, corresponding to a vasogenic edema. No contrast enhancement was proved. At this moment, the final diagnosis was unilateral atypical PRES. She was followed up during one year and there were no new symptoms or sequelae.

## 3. Discussion

### 3.1. Epidemiology

Posterior reversible encephalopathy syndrome (PRES) is a clinical-radiological entity that was well described by Hinchey et al. [1] in 1996 based on 15 cases. Its global incidence is unknown [4]. But there are a lot of scientific publications about it. Of course, since 1996, there has been a range of more than 1000 publications describing its diverse etiology, variety of clinical symptoms, and typical neuroradiological features [5].

### 3.2. Pathophysiology

The pathophysiology of PRES remains controversial. The two main hypotheses contradict each other. One involves impaired cerebral autoregulation responsible for an increase in cerebral blood flow (CBF), whereas the other involves endothelial dysfunction with cerebral hypoperfusion [6]. This hypoperfusion hypothesis may be most relevant to cases of PRES associated with cytotoxic therapy. Under both hypotheses, the result of the cerebral blood perfusion abnormalities is blood-brain barrier dysfunction with cerebral vasogenic edema [3].

#### 3.2.1. Typical Pattern of PRES

Typically, PRES presents as widespread, symmetrical, and bilateral, usually reversible vasogenic edema, cortical and subcortical, which backs up into the deep white matter as it becomes more severe [6, 7]. Predominant locations are the occipital and parietal lobes [1]. In large retrospective studies, this is the most common pattern of edema [6–9]. In addition to the occipital and parietal symmetric pattern of vasogenic edema, wide variations of distribution have been described in the literature.

### 3.3. Atypical Pattern in Our Cases

#### 3.3.1. Atypical Distribution: Unilateral Form

Although typically symmetrical and bilateral, asymmetrical or unilateral distributions of vasogenic edema in PRES can also be seen. This is designed as “tumefactive PRES” by McKinney et al. [6]. That is the situation in our case. The problem of this situation is that confusion can exist with a space occupying lesion. Patel et al. reported the same situation in one case [10], where a plain head CT imaging revealed the presence of a space occupying lesion and surrounding vasogenic edema in the left occipital lobe. Grey matter around the edema gave the impression of a space occupying lesion. A preliminary diagnosis of a space occupying primary malignant tumor lesion was made on initial presentation following CT imaging. However, this was revised after follow-up MRI, to PRES. The main risk in this situation is an unnecessary and contraindicated biopsy.

#### 3.3.2. Atypical Initial Presentation: Cerebral Hemorrhage, Focal Hematomas

Our first case has been diagnosed with an intracerebral haemorrhagic complicated form of PRES. The aspect was bilateral multiple focal hematomas. Cerebral hemorrhage is a potential complication that is known to occur in PRES. Hemorrhage is said to be an uncommon complication in PRES (5% [11] to 17% [6] of patients), based on older sequences such as FLAIR and T2^*∗*^, but it is antiquated relative to SWI (susceptibility-weighted imaging) and other newer and postprocessed sequences. The incidence of hemorrhage (particularly microhemorrhage) is higher; its frequency is more than 50%; McKinney et al. found 58.1% at presentation and 64.7% at follow-up [12]. The pathological mechanism of bleeding in PRES is still not well understood but is thought to relate to hypertension and hyperperfusion or vasculopathy with resulting hypoperfusion. Varying patterns of hemorrhage have been identified, including large focal hematomas, subarachnoid hemorrhage, or multiple minute foci of hemorrhage [8]. The differential diagnosis in this case was with cerebral metastasis of cholangiocarcinoma. But brain metastasis secondary to cholangiocarcinoma is exceedingly rare, with only a few cases reported in the literature [13, 14]. In addition, there is no hemorrhagic brain metastasis of cholangiocarcinoma reported.

### 3.4. Retrospective Diagnosis of PRES

In all of our two cases, the diagnostic of PRES has been made after partial or complete regression of the initial clinical and radiological abnormalities. In some cases, the diagnosis of PRES remains in doubt. In this situation, regression of the clinical and radiological abnormalities with appropriate treatment supports the diagnosis. Thus, repeated brain imaging is helpful [4].

## 4. Conclusion

The posterior reversible encephalopathy syndrome is a rare radioclinical syndrome. This diagnosis is easy when the clinical and radiological presentation is typical. In atypical forms, it poses the problem of differential diagnosis, which can be confused with a primary brain tumor or brain metastasis. It is often the evolution that allows rectifying the diagnosis.
